# On Prof. Esmarch’s Mode of Performing Bloodless Operations

**Published:** 1874-02

**Authors:** William MacCormac

**Affiliations:** Surgeon to St Thomas’s Hospital


### On Professor Esmarch's Mode of Performing Bloodless Operations.

By William MacCormac, Surgeon to St Thomas's Hospital,

The importance of the simple and efficient method briefly described by professor Esmarch at the second Surgical Congress in Berlin, for preventing loss of blood during operations on the limbs, is so great that Mr. MacCormac takes the earliest opportunity of communicating his experience on the subject, brief though it be.

A little girl, five years old, struck the left tibia twelve months ago against a stone; necrosis followed, and when admitted to hospital a year afterwards a sequestrum could be felt in the tibia inclosed by a considerable thickness of new bone. Whilst the patient was being chloroformed, Mr. McCormac applied pretty tightly an ordinary elastic bandage from the toes to the middle of the thigh. The bandage was two inches wide and five yards in length, and thus applied, the bandage forced all, or nearly all, the blood from the limb into the body. When the patient was fully narcotized, a half-inch India-rubber rope was wound around the thigh immediately at the upper border of the bandage, and sufficiently tight to obstruct all the afferent vessels. Hooks previously attached to the extremeties of the rope furnished a ready means of fastening it, as well as of removing it at pleasure. The bandage first applied was now unrolled, when the limb presented a blanched appearance. The operation was then commenced; some new bone removed, so as to get at and take away a considerable-sized sequestrum. During the entire time not a single drop of blood appeared in the wound; a sponge was not once required, and the facility with which the operation was conducted and finished requires to be seen to be realized. The tissues were divided, so far as bleeding was concerned, just as they might have been on the dead body. This operation was performed in St. Thomas's Hospital on August 16th in the present year, Esmarch's method for producing local anæmia being then practiced for the first time in Britain. Since the operation the little patient has progressed very favorably, and although carefully watched, no peculiarity which might be attributed to the use of the apparatus has been observed either in the wound or in the limb.

Since then other operations for necrosis have been performed, and an excision of the knee lasting thirty-five minutes, also an amputation of the thigh, and in no instance has one single drop of blood been lost. The advantages of such a plan Mr. MacCormac writes, are so palpable as not to need much insisting upon. The generality of hospital patients can ill spare a serious loss of blood, and such a loss often proves inevitable during operations for extensive necrosis of bone. In amputations the greater part of the blood of the lost extremity is preserved, to the advantage of the patient. The duration of operations will be much shortened, as there is neither blood nor the constant dabbing of sponges into the wound to remove it to interfere with the surgeon's sight; No accident or ill consequence at all appears to follow the use of the apparatus. In cases where amputation require to be performed for gangrene, or where there is a deposit of septic material in the limb about to be operated upon, there might be a risk of the elastic bandage forcing some portion of the septic material into the circulation. In the further use of the apparatus this possibility must be kept in view. Any one will be surprised, in trying it upon his own arm, to find what a small amount of pressure of the India-rubber rope will stop the pulsation of the radial artery, and the femoral can also be stopped with no great exercise of force. Doubtless the history of surgery abounds with many attempts to empty limbs of blood previous to amputation, and to arrest hemorrhage during their performance. Stromeyer, in 1853, as he remarks in his "Maxims," adopted a plan precisely similar in principle in an operation on a brachial aneurism He bandaged the limb to a point just above the aneruism, and then applied a tourniquet. The loss of blood was very small during the operation. Billroth mentions that when he was assistant to Von Langenbeck, in 1853 and 1854, a somewhat similar plan was tried in the clinique in Berlin. Vanzetti, of Padua, relates in the *Italian Medical Gazette* that Mr. Silvestri, in Vicenza, has employed bandaging and the and the India-rubber rope compression above it in amputation, but notwithstanding, to Professor Esmarch must be attributed the credit of devising and making known a most simple, practicable, and efficient plan for wholly preventing loss of blood during operations, of whatever kind, when performed upon the extremeties of the body.—*Med. Times and Gaz.—Half Yearly Abstract.*

